# Defensins Potentiate a Neutralizing Antibody Response to Enteric Viral Infection

**DOI:** 10.1371/journal.ppat.1005474

**Published:** 2016-03-02

**Authors:** Anshu P. Gounder, Nicolle D. Myers, Piper M. Treuting, Beth A. Bromme, Sarah S. Wilson, Mayim E. Wiens, Wuyuan Lu, André J. Ouellette, Katherine R. Spindler, William C. Parks, Jason G. Smith

**Affiliations:** 1 Department of Microbiology, University of Washington, Seattle, Washington, United States of America; 2 Department of Comparative Medicine, University of Washington, Seattle, Washington, United States of America; 3 Institute of Human Virology and Department of Biochemistry and Molecular Biology, University of Maryland School of Medicine, Baltimore, Maryland, United States of America; 4 Department of Pathology and Laboratory Medicine, Keck School of Medicine of the University of Southern California, USC Norris Cancer Center, Los Angeles, California, United States of America; 5 Department of Microbiology and Immunology, University of Michigan, Ann Arbor, Michigan, United States of America; 6 Department of Medicine, Cedars-Sinai Medical Center, Los Angeles, California, United States of America; University of Alabama at Birmingham, UNITED STATES

## Abstract

α-defensins are abundant antimicrobial peptides with broad, potent antibacterial, antifungal, and antiviral activities *in vitro*. Although their contribution to host defense against bacteria *in vivo* has been demonstrated, comparable studies of their antiviral activity *in vivo* are lacking. Using a mouse model deficient in activated α-defensins in the small intestine, we show that Paneth cell α-defensins protect mice from oral infection by a pathogenic virus, mouse adenovirus 1 (MAdV-1). Survival differences between mouse genotypes are lost upon parenteral MAdV-1 infection, strongly implicating a role for intestinal defenses in attenuating pathogenesis. Although differences in α-defensin expression impact the composition of the ileal commensal bacterial population, depletion studies using broad-spectrum antibiotics revealed no effect of the microbiota on α-defensin-dependent viral pathogenesis. Moreover, despite the sensitivity of MAdV-1 infection to α-defensin neutralization in cell culture, we observed no barrier effect due to Paneth cell α-defensin activation on the kinetics and magnitude of MAdV-1 dissemination to the brain. Rather, a protective neutralizing antibody response was delayed in the absence of α-defensins. This effect was specific to oral viral infection, because antibody responses to parenteral or mucosal ovalbumin exposure were not affected by α-defensin deficiency. Thus, α-defensins play an important role as adjuvants in antiviral immunity *in vivo* that is distinct from their direct antiviral activity observed in cell culture.

## Author Summary

Mammals express abundant antimicrobial peptides, including α-defensins, to protect their epithelial surfaces from microbes. α-defensins are potently antibacterial and antiviral *ex vivo*; however, their contribution to host defense from viral infection *in vivo* has not been demonstrated. We show that mice lacking functional α-defensins in their small intestines are more susceptible to disease caused by oral viral infection. Although the virus is sensitive to α-defensin antiviral activity in cell culture, the protective effect of α-defensins *in vivo* is due to a neutralizing antibody response to the virus that is delayed when α-defensins are absent. Thus, α-defensins play an important role as adjuvants in antiviral immunity *in vivo* that is distinct from their direct antiviral activity observed in cell culture.

## Introduction

In addition to a sophisticated adaptive immune system, mammals retain more primitive immune effectors, such as antimicrobial peptides, as components of the innate response to microbial infection. In humans, one of the most abundant classes of antimicrobial peptides is α-defensins [1, 2]. α-defensins are subdivided into myeloid α-defensins [e.g., human neutrophil peptides (HNP) 1–4], expressed primarily in neutrophils and certain other immune cells, and enteric α-defensins [e.g., human defensins (HD) 5 and 6], expressed by specialized Paneth cells in the small intestinal epithelium and by epithelial cells in the genitourinary tract. α-defensins have potent antiviral and antibacterial activities *in vitro* and in cell culture against a wide range of organisms. Although the multifaceted contribution of α-defensins to shaping the composition of the ileal bacterial commensal microbiota and to defense against multiple enteric bacterial pathogens *in vivo* has been described, comparable studies of α-defensin antiviral activity *in vivo* are lacking [3]. Moreover, clinical correlations between defensin abundance and viral transmission or disease are not clear [2]. To address this gap in knowledge, we investigated mouse adenovirus type 1 (MAdV-1) pathogenesis in mice lacking functional enteric α-defensin processing, the matrix metalloproteinase-7 knockout (*Mmp7* ^*-/-*^) mouse, as a system in which to study a viral pathogen in its natural host.

The *Mmp7* ^*-/-*^ model provides an elegant solution to overcome the complexity of creating a genetic α-defensin knockout mouse. Mice lack myeloid α-defensins, as all putative myeloid α-defensin genes in the genome have been converted to pseudogenes; however, there has been an expansion of the locus encoding enteric α-defensins, also known as cryptdins [4]. The Defa gene cluster spans ~ 0.8 Mbp and is interspersed with non-defensin genes [4, 5]. Although there are many cryptdin isoforms, they all share the requirement for a common proteolytic processing enzyme to convert inactive pro-α-defensin forms to active, mature forms within Paneth cells [6, 7]. This step is mediated by MMP7. Accordingly, *Mmp7* ^*-/-*^ mice lack functional α-defensins in ileal Paneth cells, and they are α-defensin deficient in the ileal lumen [8]. Because *Mmp7* is not expressed in the intact epithelium of any tissue in unchallenged mice other than Paneth cells and efferent ducts of the adult male reproductive tract [9–12], the effect of the *Mmp7* deletion is functionally Paneth cell-specific in the gut in the naïve mouse. Thus, *Mmp7* ^*-/-*^ mice provide a rational model for dissecting the role of α-defensins in enteric defense of bacterial [6, 8, 13] and viral pathogenesis.

MAdV-1 pathogenesis has been studied in some detail [14]. Upon parenteral infection, the virus disseminates in the mouse, with particular tropism for macrophages and endothelial cells and high viral loads in the brain and spleen. Mice lacking B cells are much more sensitive to acute infection, establishing a protective role for neutralizing antibodies (NAbs) [15]. T cells contribute to immunopathology of acute infection but are instrumental in controlling and ultimately clearing infection [16]. MAdV-1 pathogenesis in adult mice is typified by encephalitis, as the virus is able to cross the blood-brain barrier and stimulate inflammation [17, 18]. In this study, we compared oral MAdV-1 infection in wild type and *Mmp7* ^*-/-*^ mice and observed increased sensitivity of *Mmp7* ^*-/-*^ mice to viral disease. This is consistent with the ability of both human and mouse α-defensins to neutralize infection by MAdV-1 in cell culture. Nonetheless, the kinetics of viral dissemination out of *Mmp7* ^*-/-*^ and wild type mouse intestine were similar, inconsistent with a direct antiviral barrier due to the presence of α-defensins in wild type but not *Mmp7* ^*-/-*^ mouse intestine. Rather, we observed decreased viral loads in multiple organs of wild type mice compared to *Mmp7* ^*-/-*^ mice only at late times post-infection (p.i.) that were coincident with the elaboration of a robust NAb response in wild type but not *Mmp7* ^*-/-*^ mice. Thus, our data support an antiviral role for α-defensins through potentiating a NAb response. This mechanism is distinct from direct α-defensin antiviral activity at the site of initial infection.

## Results

### MAdV-1 is sensitive to α-defensin neutralization

**Fig 1:**
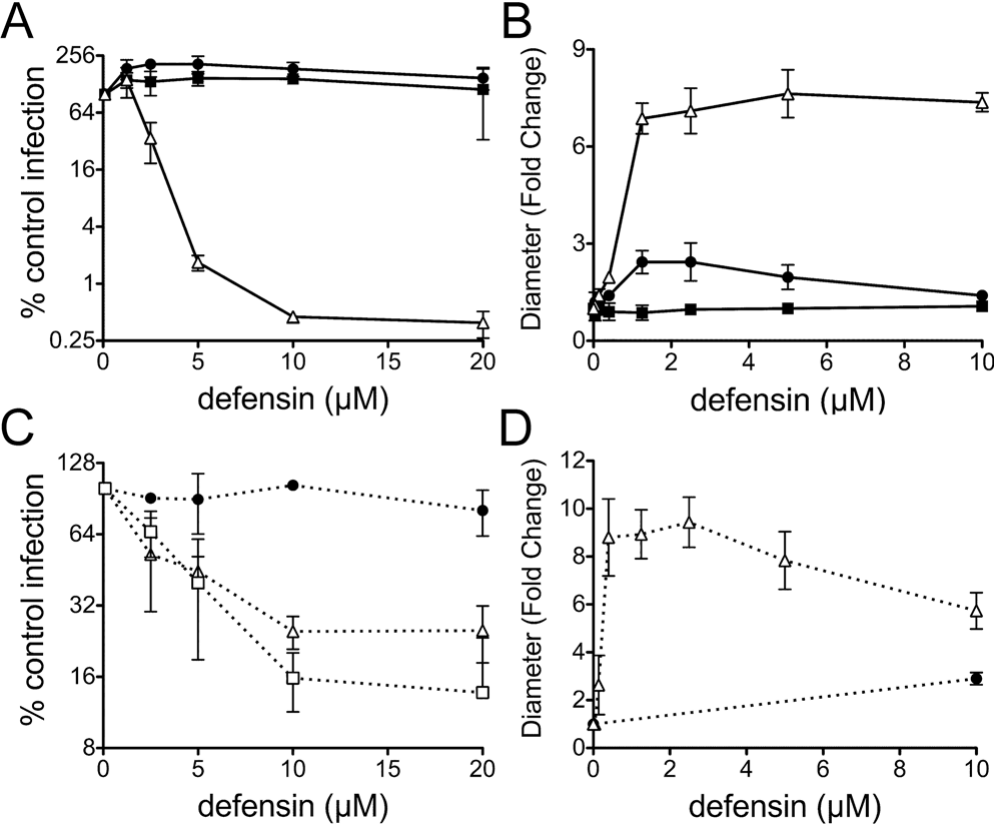
Defensins bind to MAdV-1 and potently neutralize infection *in vitro*. **(** *A* and *C*) Infection of CMT-93 cells by MAdV-1.IXeGFP pre-incubated with the indicated concentrations of α-defensins is expressed relative to control cells infected in the absence of α-defensin (100%). Values are the means of at least three independent experiments ± SD (*A*) for HD5 (open triangle), proHD5 (filled circle), and HD5Abu (filled square) and (*C*) for Crp2 (open triangle), Crp4 (filled circle), and Crp23 (open square). (*B* and *D*) The fold change in z-average diameter generated from cumulant analysis of dynamic light scattering of wild type MAdV-1 incubated with increasing concentrations of α-defensins over that of virus alone. Symbols in *B* are as in *A;* symbols in *D* are as in *C*. Data are the means of at least three independent experiments ± SD.

To establish a model to assess the role of α-defensins in adenoviral pathogenesis, we first determined the sensitivity of MAdV-1 infection in cell culture to neutralization by mouse and human α-defensins. Since mice lack neutrophil α-defensins but express abundant enteric α-defensins [19], we focused our studies on the human enteric α-defensin HD5 and three specific isoforms of mouse α-defensins, cryptdins 2, 4, and 23 (Crp2, Crp4, and Crp23). We used a replication-competent MAdV-1 construct expressing and encapsidating a fusion protein between eGFP and the minor capsid protein IX (MAdV-1.IXeGFP) to facilitate quantification of viral infection. Upon incubation of purified MAdV-1.IXeGFP with physiologic concentrations of purified α-defensins, we observed dose-dependent neutralization by HD5, Crp2, and Crp23 (Fig 1). Although the 50% inhibitory concentrations (IC_50_s) for all of these α-defensins were similar (~2.5 μM), HD5 was the most potent peptide and capable of nearly complete inhibition (Fig 1*A*). Crp2 and Crp23, which differ by a single amino acid, had almost identical activities and inhibited infection ~4-fold (Fig 1*C*). In contrast, Crp4 had no detectable antiviral activity even at the highest concentrations tested (Fig 1*C*). Although Crp4 is potently anti-bacterial *in vitro*, it has an unusual internal 3 amino acid deletion that may contribute to its inability to inhibit MAdV-1 [20]. In addition, we observed no antiviral activity for HD5Abu, which lacks a regular structure due to the absence of disulfide bonds (Fig 1*A*). We then measured viral aggregation as a function of α-defensin concentration. We have previously shown that aggregation is directly correlated with the ability of α-defensins to bind to virus but is not sufficient for neutralization [21]. We found a dose-responsive increase in the mean particle size (z-average diameter) of MAdV-1 with increasing concentrations of HD5 and Crp2, consistent with viral aggregation (Fig 1*B* and 1*D*). This was not observed for Crp4 or HD5Abu. Thus, human and mouse α-defensins potently block MAdV-1 infection by binding to the virus in a manner dependent upon their disulfide-stabilized structures.

### MMP7-null mice are more susceptible to oral infection by MAdV-1

**Fig 2:**
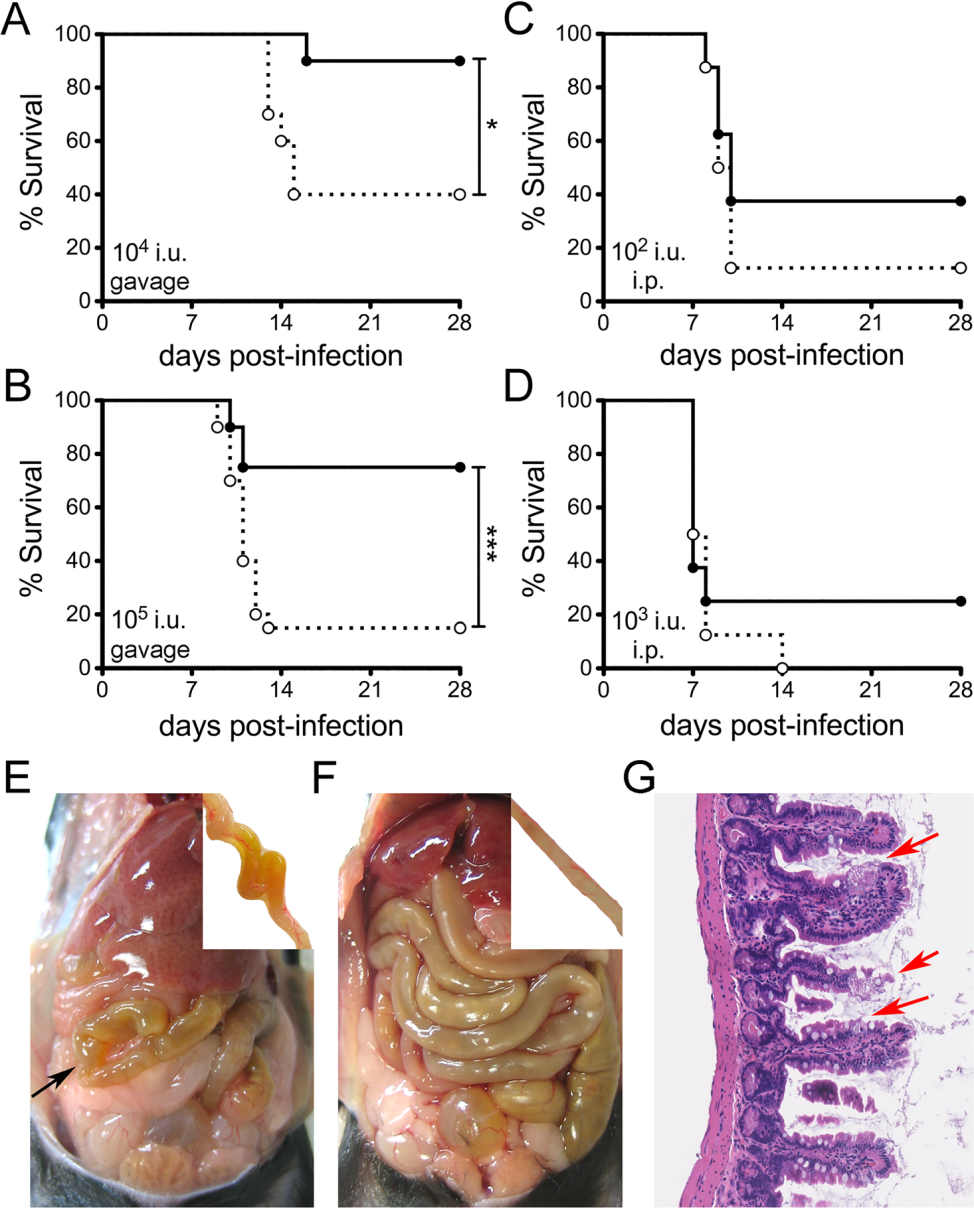
Reduced survival of *Mmp7* ^*-/-*^ mice upon oral but not parenteral infection. Survival curves of wild type (black circle and solid line) and *Mmp7* ^*-/-*^ mice (open circle and dotted line) infected (*A* and *B*) by oral gavage or (*C* and *D*) intraperitoneally (i.p.) with MAdV-1 as follows: (*A*) 1x10^4^ i.u. (n = 10), (*B*) 1x10^5^ i.u. (n = 20, combined from two independent experiments), (*C*) 1x10^2^ i.u. (n = 8), and (*D*) 1x10^3^ i.u. (n = 8). *P<0.05, ***P<0.001, unmarked curves were not significant (P>0.05). (*E* and *F*) Gross anatomy of representative *Mmp7* ^-/-^ (*E*) and wild type (*F*) mice 11 d p.i. with 1x10^5^ i.u. of MAdV-1 by oral gavage. Arrow in (*E*) points to a yellow discolored segment of bowel. Insets show a yellow discolored segment of small bowel (*E*) or normal bowel (*F*) from these mice after flushing the lumen with PBS. (*G*) Representative H&E-stained section at 20× magnification from an *Mmp7* ^*-/-*^ mouse with a yellowish, dilated small intestine showing an area with villus blunting and death of enterocytes (arrows) adjacent to intact cells.

All mouse α-defensin precursors (pro-α-defensins) are processed to mature forms within Paneth cells by MMP7 proteolysis. Accordingly, *Mmp7* ^-/-^ mice lack activated α-defensins, and their Paneth cells secrete pro-α-defensins [6, 8, 13]. Pro-α-defensins, exemplified by proHD5, did not bind to MAdV-1 or block infection *in vitro* (Fig 1*A* and 1*B*). To directly test the impact of α-defensin antiviral activity on viral pathogenesis *in vivo*, we infected wild type C57BL/6 mice and isogenic *Mmp7* ^-/-^ mice with MAdV-1 by oral gavage. Consistent with the sensitivity of MAdV-1 to inhibition by α-defensins in cell culture, *Mmp7* ^-/-^ mice succumbed in greater numbers to MAdV-1 infection (Fig 2*A* and 2*B*). This phenotype was dose-dependent. Yellow discolorations of the small intestine were noted at necropsy for both wild type (5 of 6) and *Mmp7* ^-/-^ (20 of 24) mice euthanized due to illness (Fig 2*E*). The affected areas were often discontinuous, although in some cases almost the entire small intestine was involved, and typified by diffuse villus blunting (Fig 2*G*), while the caecum and colon appeared normal. Overall, the phenotypes of sick wild type and *Mmp7* ^-/-^ mice were similar, with noticeable bowel discoloration; however, the frequency of sick mice was substantially higher among *Mmp7* ^-/-^ mice, and most wild type mice at a comparable time p.i. had no obvious lesions (Fig 2*F*).

In unchallenged wild type mice, Paneth cells are the only cells in the intestine that produce MMP7 [22]. Thus, we challenged mice intraperitoneally (i.p.) to determine whether the effect of the MMP7 deletion is localized to the intestine. Unlike oral infection, survival from parenteral challenge was equivalent for both mouse genotypes at two different MAdV-1 doses (Fig 2*C* and 2*D*). These findings support the interpretation that a major role for MMP7 in protection from oral viral infection is at the initial site of infection in the small bowel.

### Depletion of intestinal commensals does not alter the susceptibility of wild type or *Mmp7* ^-/-^ mice to oral infection by MAdV-1

**Fig 3:**
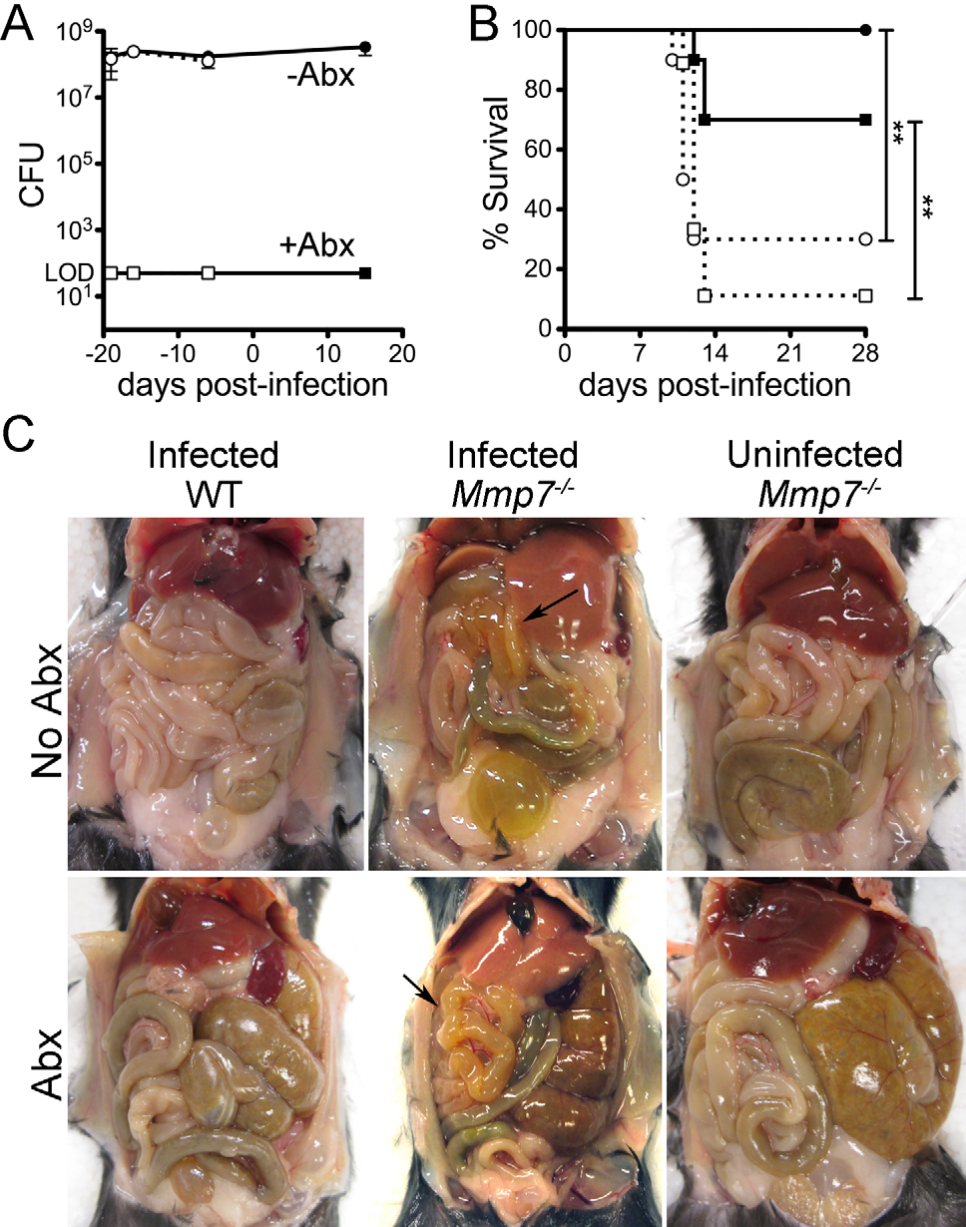
*Mmp7* ^*-/-*^ mice depleted of commensals maintain increased susceptibility to MAdV-1. (*A*) Feces from antibiotic treated (squares) or untreated control mice (circles) from wild type (solid symbols) or *Mmp7* ^*-/-*^ mice (open symbols) were cultured anaerobically. Data are mean viable colony forming units (CFU) from 2–5 mice per condition per time point ± SD. The limit of detection (LOD) was 50 CFU. (*B*) Survival curves of antibiotic treated and control mice challenged with 9x10^5^ i.u. of MAdV-1 by oral gavage (n = 10 for all except *Mmp7* ^*-/-*^ mice plus antibiotics for which n = 9). Symbols and lines are as in (*A*). **P = 0.001–0.01, unmarked curves were not significant (P>0.05). (*C*) Gross anatomy of representative mice treated with or without antibiotics. Uninfected *Mmp7* ^*-/-*^ control mice and infected wild type mice were euthanized 28 days post-gavage. Infected *Mmp7* ^*-/-*^ mice were euthanized due to illness 10 days (no Abx) or 12 days (Abx) p.i. Arrows point to yellow discolored bowel segments of infected *Mmp7* ^-/-^ mice.

In addition to removing a potential direct antiviral effect of α-defensins through deletion of *Mmp7*, the absence of functional α-defensins in these mice could impact MAdV-1 infection indirectly by altering the intestinal commensal microbiota [23]. Although the overall abundance of commensals in *Mmp7* ^-/-^ mice does not differ from that of wild type mice, the composition of the ileal microbiota is changed [23]. Commensal bacteria influence infection and pathogenesis of poliovirus, reovirus, mouse mammary tumor virus, and norovirus in mouse models [24–27]. We confirmed that the abundance of culturable bacteria in feces does not differ between wild type and *Mmp7* ^*-/-*^ mice (Fig 3*A*). To determine if the abundance of commensal bacteria affects MAdV-1 infection, we treated mice with a combination of ampicillin, neomycin, vancomycin, and metronidazole to deplete the intestinal microbiota of wild type and *Mmp7* ^-/-^ mice, using a previously described protocol [24, 28]. As in prior studies, treatment with antibiotics was effective, since we were unable to culture anaerobic bacteria from the feces of these mice when periodically evaluated over 39 d of continuous antibiotic treatment (Fig 3*A*). We then assessed survival and weight change for both antibiotic-treated and mock-treated control mice upon challenge with MAdV-1 by oral gavage. As in our previous experiments (Fig 2), *Mmp7* ^-/-^ mice were more susceptible to MAdV-1 infection (30% survival) compared to wild type mice in the absence of antibiotic treatment (100% survival, Fig 3*B*). Antibiotic treatment resulted in transient weight loss in mice of both genotypes and greater variability in daily weight change compared to untreated mice (S1 Fig). Nonetheless, survival differences within genotypes between the antibiotic-treated and control mice were not significant, but a significant survival difference between the antibiotic-treated *Mmp7* ^-/-^ (11% survival) and wild type (70% survival) mice was still observed (Fig 3*B*). Thus, unlike the case for some other viral pathogens, the abundance of commensals did not appear to impact MAdV-1 pathogenesis upon oral infection. In addition, yellowish discoloration of the small bowel was also observed in sick untreated and antibiotic treated mice (7 of 8 *Mmp7* ^-/-^ and 2 of 3 wild type) (Fig 3*C*). From these experiments, we conclude that alterations in the composition of the commensal microbiota due to differential expression of *Mmp7* are unlikely to explain the survival difference between wild type and *Mmp7* ^-/-^ mice.

### Early kinetics of MAdV-1 dissemination following oral infection are identical in wild type and *Mmp7* ^-/-^ mice

**Fig 4:**
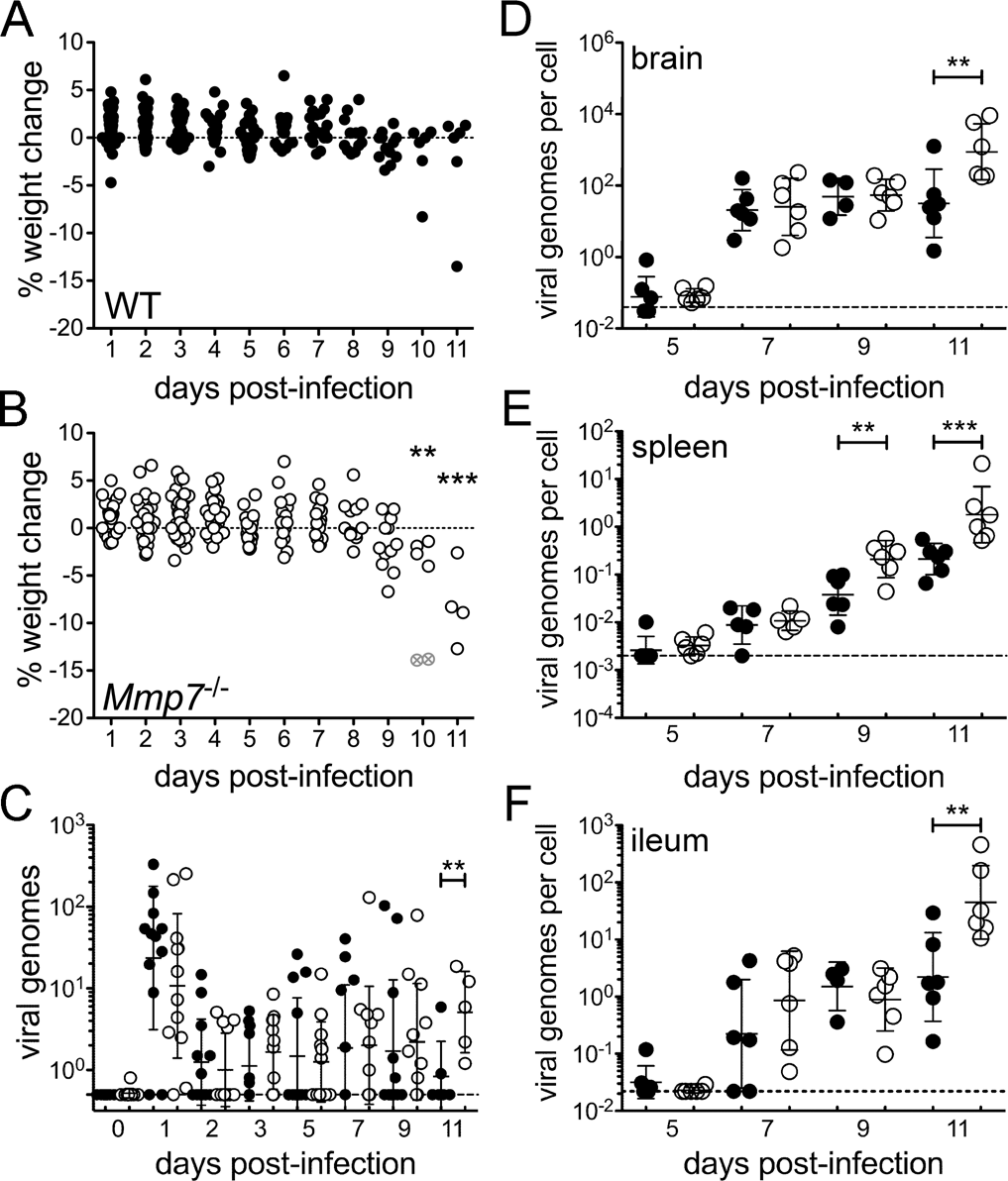
MAdV-1 loads are higher in *Mmp7* ^*-/-*^ mice than wild type only late in infection. Weight change from measure to measure for each (*A*) wild type or (*B*) *Mmp7* ^*-/-*^ mouse is shown after infection with 1x10^5^ i.u. of MAdV-1 by oral gavage. Open gray symbols containing an “x” denote the two mice that were humanely euthanized due to illness. MAdV-1 genomes in (*C*) fecal pellets or the ratio of MAdV-1 genomes to cellular genomes in (*D*) brain, (*E*) spleen, and (*F*) distal ileum were quantified by qPCR. Data are the individual values for each wild type (closed symbols) or *Mmp7* ^*-/-*^ (open symbols) mouse overlaid with the group mean ± SD (n = 6 except for fecal samples on days 0–9, where n = 12). For all graphs, **P = 0.001–0.01 and ***P<0.001 comparing wild type and *Mmp7* ^*-/-*^ mice on the same day. Unmarked days were not significant (P>0.05). Dashed lines in (*C*-*F*) indicate the limits of detection.

To examine the systemic dissemination of MAdV-1 and the development of pathology in orally infected mice in more detail, we undertook a time course study. Wild type and *Mmp7* ^-/-^ mice were infected with MAdV-1 (1x10^5^ i.u.) and monitored for weight loss, and cohorts of six mice were euthanized every other day up to day 11 p.i., the last time point before extensive mortality in *Mmp7* ^-/-^ mice would preclude further comparisons with wild type. As in previous experiments, all of the *Mmp7* ^-/-^ mice lost weight between 9 and 11 d p.i., and four of the six mice in the day 11 cohort were overtly ill, including two mice that were euthanized on day 10 (Fig 4*B*). In contrast, only one wild type mouse lost weight, and no wild type mice exhibited outward signs of illness (Fig 4*A*).

**Fig 5:**
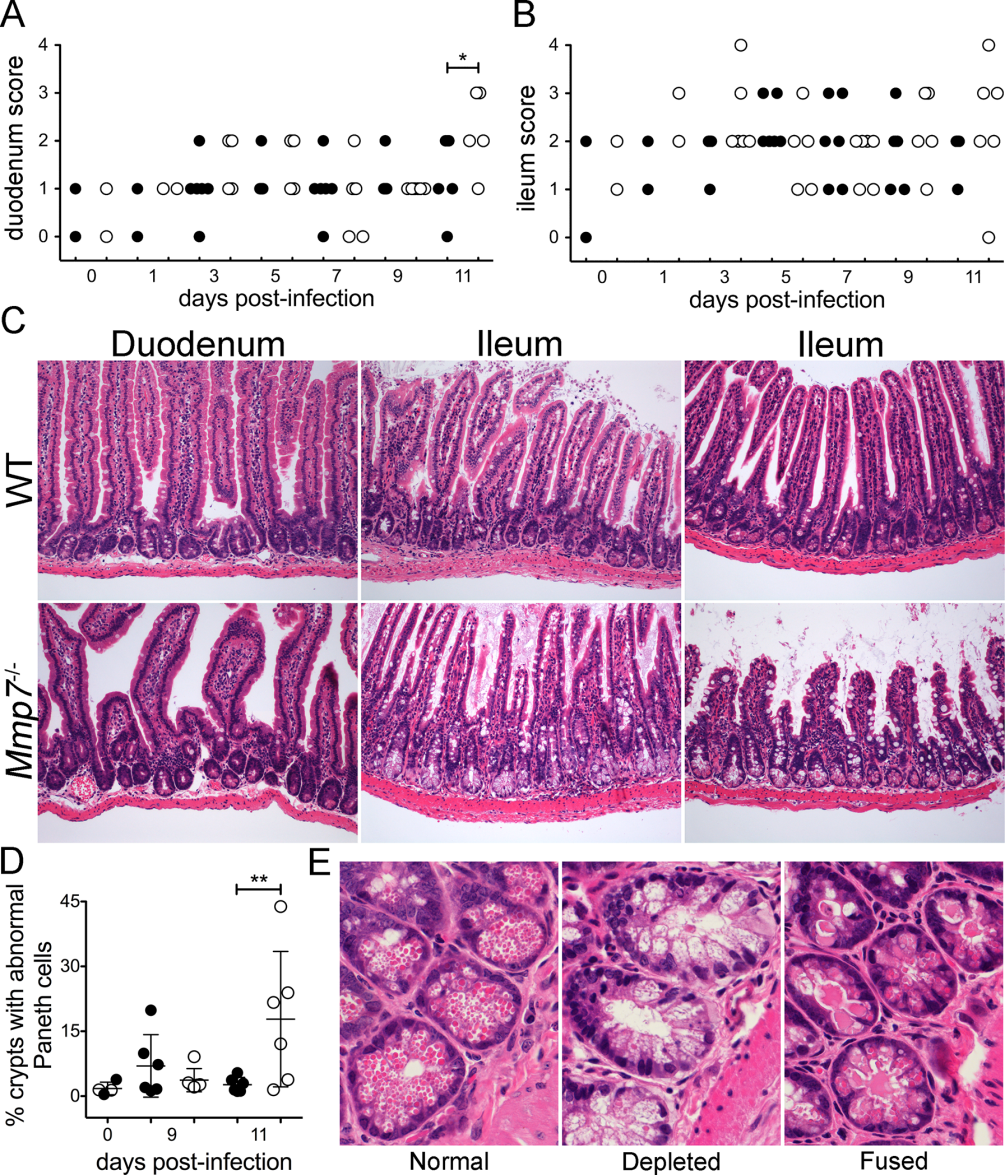
Small intestinal pathology following MAdV-1 infection. Severity of (*A*) duodenal or (*B*) ileal enteritis for the wild type (closed symbols) and *Mmp7* ^*-/-*^ (open symbols) mice from Fig 4 (n = 6 except for days 0 and 1, where n = 2) scored for the infiltration of inflammatory cells into the mucosa on the following scale: 0, none; 1, scattered cells; 2, multifocal accumulations of small clusters of 3–5 cells; 3, multifocal accumulations of mild clusters of 6–10 cells with edema; 4, 3 plus evidence of tissue damage (necrosis, apoptosis). *P<0.05, unmarked days were not significant. (*C*) Representative H&E-stained sections from infected mice of each genotype on day 11 p.i. at 20× magnification. Although sample preparation did not enable careful quantitation, apparent villus blunting equivalent to that of the lower right panel was observed in the ileums of 5 of 6 Mmp7^-/-^ mice but only 2 of 6 WT mice on day 11. (*D*) Phenotypes of Paneth cells in ileal crypts. An average of 560 crypts per mouse was assessed. Data are the percent of crypts with fused or depleted Paneth cells for each wild type (closed symbols) or *Mmp7* ^*-/-*^ (open symbols) mouse overlaid with the group mean ± SD (n = 6 except for day 0, where n = 2 of each genotype). **P = 0.001–0.01, unmarked days were not significant. (*E*) Representative H&E-stained images at 60× magnification of each crypt phenotype that was quantified in (*D*).

We quantified copies of the MAdV-1 genome in feces to monitor viral shedding (Fig 4*C*) and MAdV-1 genome copies per cellular genome in brain (Fig 4*D*), spleen (Fig 4*E*), and ileum (Fig 4*F*) to monitor viral replication and spread. In each case, we observed virtually identical viral shedding and viral loads in tissues of wild type and *Mmp7* ^-/-^ mice up to day 7 p.i., including passage of the inoculum in feces on day 1. On day 9, there was a significant increase in viral loads in *Mmp7* ^-/-^ spleens compared to wild type. On day 11, viral shedding in feces and viral loads in all organs examined were higher in *Mmp7* ^-/-^ mice than wild type. Histopathologic analysis of intestine revealed a significant difference for duodenal enteritis between genotypes on day 11 (Fig 5*A* and 5*C*). Due to a general trend towards more severe ileal enteritis in *Mmp7* ^-/-^ mice on day 11 (Fig 5*B* and 5*C*), we more closely examined the ileal crypts. Crypts with abnormal Paneth cell phenotypes, either fusion (Fig 5*E*, right panel) or complete depletion of the cytoplasmic secretory granules (Fig 5*E*, center panel), were significantly more prevalent in *Mmp7* ^-/-^ mice than wild type on day 11 but not on day 9 (Fig 5*D*). Thus, reduced survival of *Mmp7* ^-/-^ mice correlated with increased viral loads in multiple tissues and more severe lesions and more prevalent Paneth cell defects in the intestine; however, the early kinetics of viral dissemination were independent of *Mmp7* expression.

### *Mmp7* ^-/-^ mice mount a delayed NAb response to MAdV-1 despite robust antibody responses to systemic or mucosal exposure to model antigens

**Fig 6:**
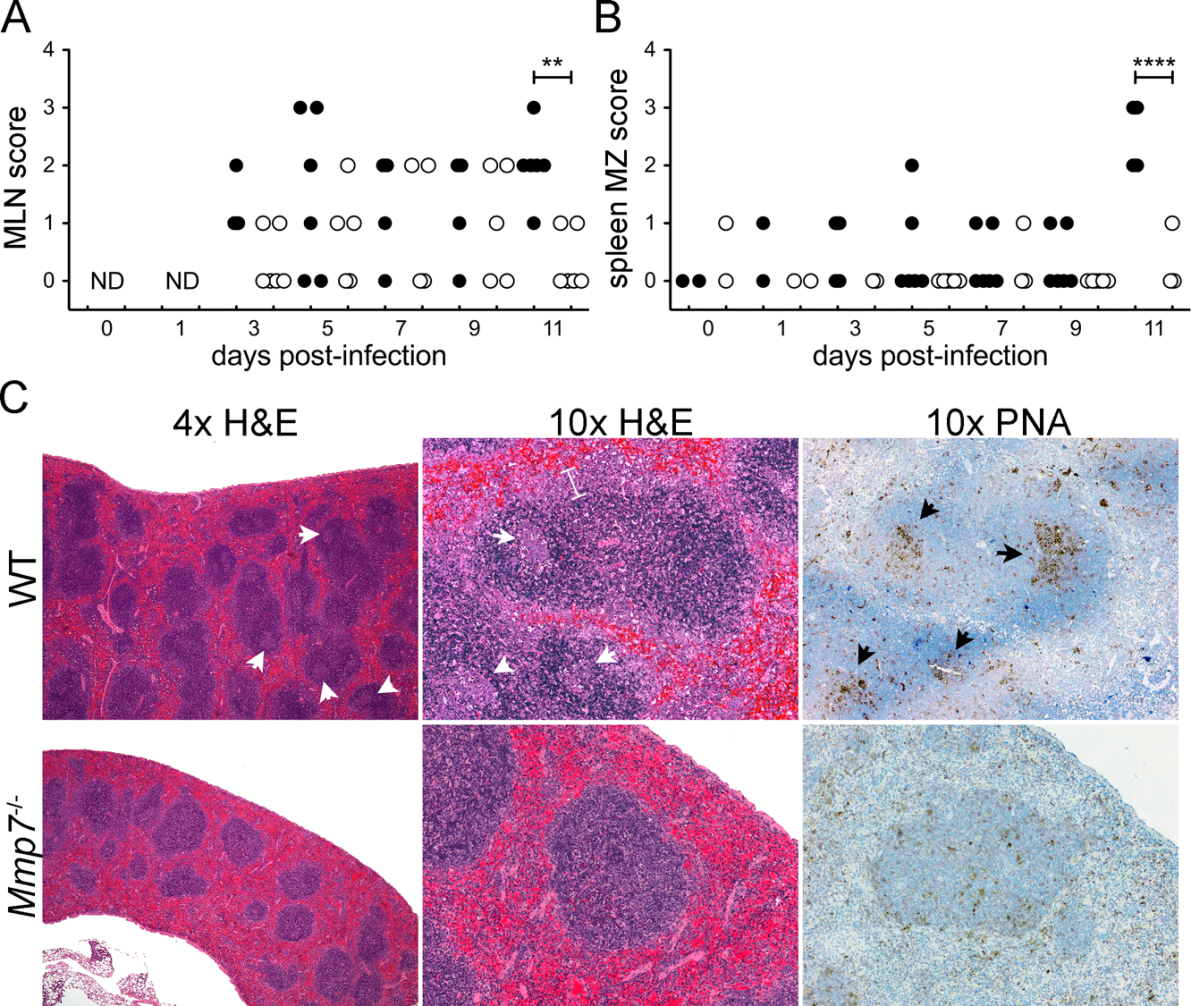
Hallmarks of adaptive immune activation in spleens and MLNs of wild type but not *Mmp7* ^*-/-*^ mice. For the same wild type (closed symbols) and *Mmp7* ^*-/-*^ (open symbols) mice as in Fig 4, (*A*) MLN follicular hyperplasia was scored on the following scale: 0, none; 1, minimal diffuse increase in cellularity; 2, mild enlargement with 1–2 small germinal centers; 3, >2 germinal centers. (*B*) Splenic marginal zone hyperplasia was scored on the following scale: 0, none; 1, 1.5× normal; 2, 2× normal and readily observable at 4× magnification; 3, 3× normal; 4, >3× normal. (*C*) Representative H&E and peanut agglutinin (PNA) stained sections from infected mice of each genotype on day 11 p.i. at 4x and 10x magnification. PNA stained sections are counterstained with hematoxylin. Examples of prominent germinal centers (arrowheads) and thickened marginal zone (bracket) in the wild type sample are marked. **P = 0.001–0.01 and ****P<0.0001.

**Fig 7:**
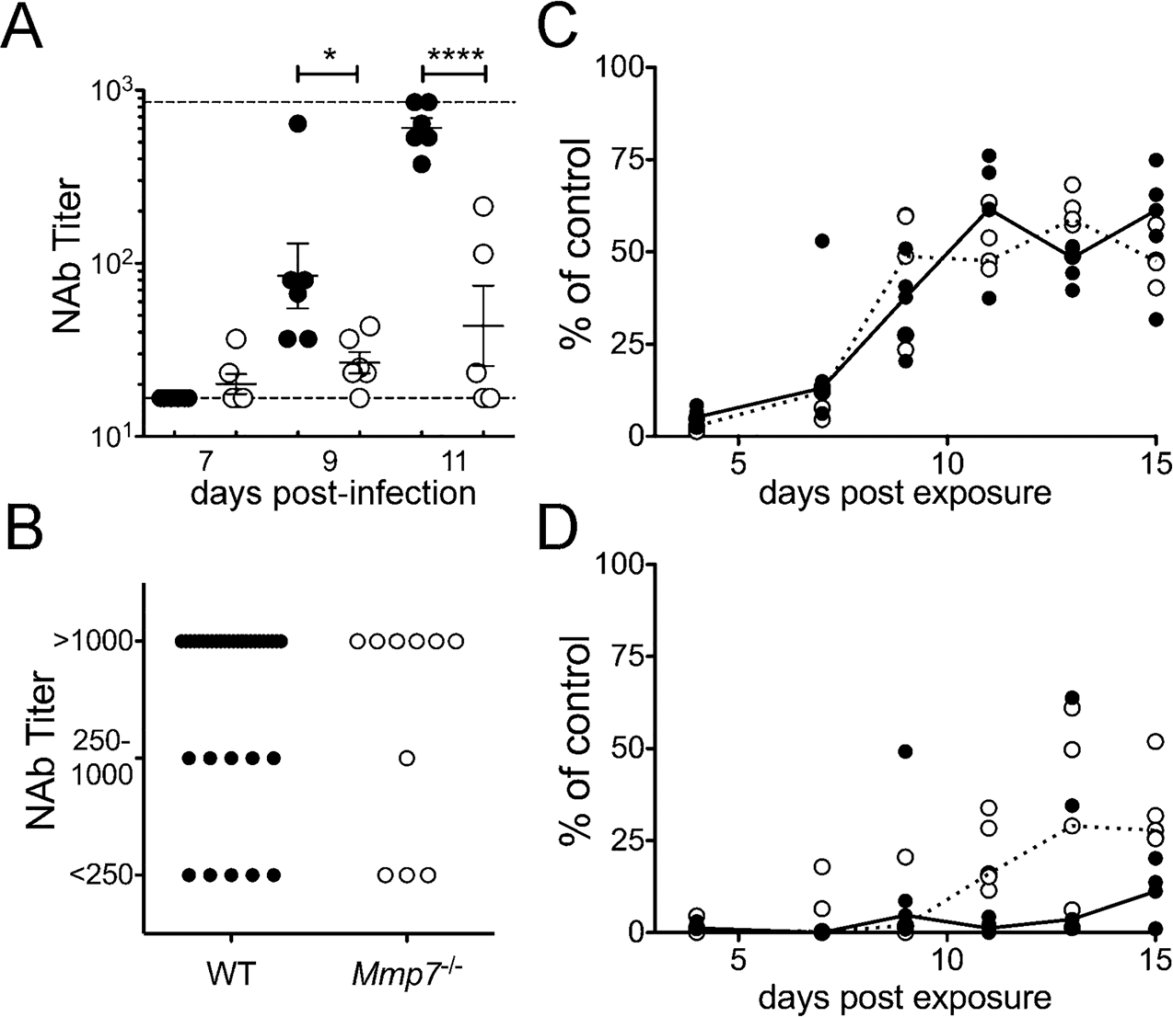
Wild type but not *Mmp7* ^*-/-*^ mice mount a protective humoral response to MAdV-1 infection. (*A*) For the same wild type (closed symbols) and *Mmp7* ^*-/-*^ (open symbols) mice as in Fig 4, serum NAb titers overlaid with the group mean ± SEM (n = 6 except for *Mmp7* ^*-/-*^ day 11, where n = 5). Upper and lower limits of detection are indicated by dashed lines. (*B*) Serum NAb titers on day 28 p.i. from mice that survived MAdV-1 infection in experiments from Figs 2 and 3 (WT, n = 38 and *Mmp7* ^*-/-*^, n = 10). (*C* and *D*) Ovalbumin-specific antibody responses from wild type (closed symbols) and *Mmp7* ^*-/-*^ (open symbols) mice inoculated with OVA+LPS i.p. (*C*) or intranasally (*D*) relative to a hyperimmune control (n = 5 per treatment). Lines connect median values for each group. *P<0.05 and ****P<0.0001. Differences between genotypes in *B*-*D* are not significant (P>0.05).

If direct antiviral activity of mature α-defensins was responsible for the reduced survival of *Mmp7* ^-/-^ mice, we would expect to see delayed or reduced early kinetics of viral dissemination in wild type mice compared to *Mmp7* ^-/-^ mice. Since this was not observed, we hypothesized that α-defensins were modulating pathogenesis indirectly. One clue to the mechanism arose from our analysis of the spleens and mesenteric lymph nodes (MLNs) of wild type and *Mmp7* ^-/-^ mice. MLN follicular hyperplasia and splenic marginal zone hyperplasia were significantly increased in wild type mice compared to *Mmp7* ^-/-^ mice on day 11 p.i. (Fig 6*A* and 6*B*) and were the most striking histologic differences between infected mice of the two genotypes. Moreover, germinal centers were readily apparent even at low magnification (4x) by H&E (pale staining regions in the follicles) and peanut agglutinin (PNA) staining (brown staining regions in the follicles) in the spleens of wild type mice, whereas they were not distinct even at high magnification in splenic sections from *Mmp7* ^-/-^ mice (Fig 6*C*). Collectively, these histologic findings suggest a robust adaptive immune response to MAdV-1 in wild type mice that is absent or delayed in *Mmp7* ^-/-^ mice. To test this directly, we determined the NAb titers of sera from mice of both genotypes. All wild type mice developed detectable serum NAbs by day 9 p.i., which were increased in titer on day 11 (Fig 7*A*). In contrast, little neutralizing activity was observed in sera from *Mmp7* ^-/-^ mice on day 9, and on day 11 only two of five *Mmp7* ^-/-^ mice had measureable NAb titers. From these data, we conclude that the inability of *Mmp7* ^-/-^ mice to mount a timely protective NAb response to MAdV-1 infection explains the reduced survival in these mice after viral challenge.

To determine whether the humoral response in *Mmp7* ^-/-^ mice is globally compromised, we exposed mice of both genotypes systemically (via i.p. injection) or mucosally (via intranasal instillation) with ovalbumin (OVA) using lipopolysaccharide (LPS) as an adjuvant. Since oral administration of OVA may be confounded by the development of oral tolerance, we chose intranasal instillation as an established approach to stimulate a mucosal immune response [29, 30]. In contrast to responses to MAdV-1 infection, all mice of both genotypes mounted humoral responses to both systemic and mucosal antigen and with identical kinetics (Fig 7*C* and 7*D*). Also, serum NAb responses of the few *Mmp7* ^-/-^ mice that survived MAdV-1 infection in all of our experiments were similar to those of wild type mice when assayed 28 d p.i. (Fig 7*B*). Thus, the humoral response in *Mmp7* ^-/-^ mice is not globally compromised; rather the specific NAb response to enteric MAdV-1 infection is delayed.

## Discussion

The impact of α-defensins on viral transmission or pathogenesis has not been previously examined experimentally; however, correlates from clinical samples suggest that high α-defensin levels are associated with reduced transmission of HIV-1 and slower disease progression [2]. In studies of β- and θ-defensins in viral pathogenesis, the expression or administration of defensin reduced viral immunopathology without impacting viral titers [31, 32]. These studies suggest a more profound effect of defensins on limiting innate immunopathology than as direct antivirals. Similarly, despite their direct antiviral activity in cell culture, the effect of α-defensins on MAdV-1 pathogenesis appears to be indirect. Taken together, our data support a mechanism in which functional processing of enteric α-defensins at the initial site of viral infection in the small intestine is a critical modulator of the protective NAb response, which is required for survival from acute MAdV-1 infection [15]. Myeloid α-defensins (HNPs) function as adjuvants [30, 33]. When mixed with OVA and administered intranasally, HNPs increased anti-OVA serum IgG but not IgA [30]. HNPs also enhanced CD4^+^ T cell cytokine secretion and proliferation following stimulation either *in vivo* or *in vitro*, suggesting an ability of HNPs to stimulate T cell-dependent cellular and humoral immunity. This was substantiated in intraperitoneal immunization studies with other model antigens and tumor-specific antigens [33]. Enteric α-defensins, either mouse or human, have not been previously reported as adjuvants. Moreover, this is the first example of a direct role for an α-defensin in engendering an adaptive response to a pathogen.

There are several possibilities for how α-defensins could enhance the adaptive immune response to MAdV-1. First, given the capacity of α-defensins and HD5 to bind to and aggregate MAdV-1, one possibility is that an immunogenic complex of virus and defensin forms in the intestine. The aggregate could be taken up by antigen presenting cells differently than free virus, perhaps through unidentified defensin-specific receptors, in the same way that bacteria opsonized by rabbit myeloid α-defensins are more readily taken up by macrophages [34]. Alternatively, the virus-defensin complex could be potently chemotactic for immune cells, a property of some α-defensins, including HD5, which has not been reported for mouse enteric α-defensins [35, 36]. Although we found no clear evidence for a direct antiviral effect of α-defensins *in vivo*, if our model for human AdV neutralization by α-defensins also holds true for MAdV [37, 38], prolonged dwelling in the endosomal pathway during virus entry into the cell could alter exposure of the virus to innate immune sensors (e.g., toll-like receptor 9) with downstream effects on the development of adaptive immunity. Finally, there may be a direct effect of enteric α-defensins on B cell or T helper cell function. Extrapolating from our analysis of proHD5, proCrps found in *Mmp7* ^-/-^ mice are unlikely to bind to MAdV-1 to mediate any of these functions.

One limitation of the *Mmp7* ^-/-^ model is that it is a complete null mutation in all tissues; therefore, we cannot formally exclude the contribution of MMP7 functions other than activation of Paneth cell pro-α-defensins to survival differences in MAdV-1 infected mice. However, there are numerous critical observations in diverse model systems as well as from our study that support the prior use of this model specifically for studies of enteric α-defensin functions [6, 8, 13] and our interpretation that the survival phenotype in these mice is due to pro-α-defensin processing by MMP7 rather than other potential MMP7-dependent functions. First and foremost, other than Paneth cells and efferent ducts of the adult male reproductive tract, MMP7 is not expressed in the intact epithelium of other tissues in naïve mice [9–12, 22]. In addition, other than a complete lack of α-defensin processing, no alteration in cell behavior or gene expression has been observed in these mice, although global gene expression analysis has not been performed [39]. Similarly, we have found no differences in Paneth or goblet cell numbers or the morphology of small intestinal organoids from wild type and *Mmp7* ^*-/-*^ mice [13]. Thus, MMP7 deficiency is functionally Paneth cell-restricted in the intestines of unchallenged mice, and the mice are otherwise normal. Second, MMP7 is induced in epithelial cells in response to toxic or mechanical injury, bacterial infection, or oncogenic transformation, but its expression is not affected by viral infection [40–54]. When induced in response to colon or lung injury or to airway bacterial infection, *Mmp7* ^*-/-*^ mice consistently show marked protection against mortality and reduced inflammation compared to wild type mice [40–42, 55]. Here, we see just the opposite: more death (Figs 1 and 2) and greater immunopathology in the small intestine (Fig 5) of *Mmp7* ^*-/-*^ mice infected with MAdV-1 compared to wild type mice. Third, MMP7 is expressed in mice only by activated mucosal and glandular epithelia [22, 46]. Thus, direct functions of MMP7 could not account for differences in lymphoid organs observed in our experiments, as it is not expressed in lymphoid organs. Finally, upon parenteral MAdV-1 infection, we observed no survival difference between genotypes (Fig 2*C* and 2*D*), strongly implicating MMP7-dependent functions in the gut epithelium (pro-α-defensin processing in Paneth cells) rather than MMP7-dependent functions elsewhere as the primary determinant of survival differences upon MAdV-1 oral infection.

Although MMP7 deficiency affects neutrophil and CD103-positive dendritic cell efflux in the lung and pro-inflammatory cytokine (TNF-α) activation on macrophages, MMP7 deficiency has not been reported to globally attenuate adaptive immune responses [40, 42, 56]. Rather, in a model of experimental autoimmune encephalomyelitis (EAE) induced by myelin oligodendrocyte glycoprotein (MOG) exposure, splenocytes and lymphocytes from *Mmp7* ^-/-^ mice were able to respond to MOG and induce EAE in wild type mice [57]. This is consistent with our finding that MMP7 deficiency does not globally abrogate a humoral immune response, as we observe normal humoral responses to ovalbumin exposure in the nasal mucosa and upon intraperitoneal injection. Furthermore, *Mmp7* ^*-/-*^ mice that survive MAdV-1 infection have normal antibody responses when assayed 28 day p.i., (Fig 7*B*) indicating that a delayed NAb response rather than the absolute failure to mount a humoral response explains the survival difference.

In summary, our studies of a natural viral pathogen reveal a profound effect of functional α-defensins in the ileum on survival from oral infection. Although there may be some contribution of direct α-defensin antiviral activity to modulating infection, the delayed NAb response appears to be primarily responsible for the survival difference between the wild type and *Mmp7* ^-/-^ mice. Our data strongly support a role for α-defensins as adjuvants, specifically in the context of enteric viral infection; however, the exact mechanism remains to be determined. Moreover, additional studies in alternative models, ideally an α-defensin genetic knockout, are needed to formally exclude MMP7 activities beyond α-defensin maturation in engendering the antiviral adaptive immune response.

## Methods

### Viruses

Wild type MAdV-1 was originally obtained from S. Larsen (Indiana University Medical Center). MAdV-1.IXeGFP (MAV-1 inp903) was created by fusing an eGFP open reading frame (ORF) in frame 3’ to the ORF encoding capsid protein IX, using recombineering with a bacterial artificial chromosome containing the genome of MAdV-1 (pKBS2 MAV-1, a kind gift of Silvio Hemmi, University of Zurich)[58]. Wild type MAdV-1 and MAdV-1.IXeGFP were both propagated on CMT-93 mouse rectal carcinoma cells (ATCC CCL-223, a kind gift from Susan Compton, Yale University School of Medicine)[59]. CMT-93 cells were cultured in DMEM supplemented with 10% fetal bovine serum (Sigma-Aldrich), 4 mM L-glutamine, 100 units/ml penicillin, 100 μg/ml streptomycin, and 0.1 mM nonessential amino acids (complete DMEM). For *in vivo* studies, cleared tissue culture supernatant containing virus was used. For antiviral assays in cell culture and dynamic light scattering, viruses were concentrated from supernatant by polyethylene glycol precipitation and purified by CsCl gradient ultracentrifugation as described [60]. The particle concentration of purified virus was determined using a Bio-Rad Protein Assay (Bio-Rad, Hercules, CA) with a bovine serum albumin standard. The infectious titers of wild type MAdV-1 stocks were determined by infecting CMT-93 cells with serial dilutions of virus. The cells were then fixed in 2% paraformaldehyde, permeabilized with 20 mM glycine and 0.5% Triton X-100 in phosphate buffered saline (PBS), and stained with anti-hexon antibody 8C4 (Fitzgerald Industries International) and an Alexa Fluor 488-conjugated secondary antibody (Invitrogen). Hexon positive cells were enumerated by flow cytometry, and the infectious titer of the viral stock was calculated using the Poisson distribution.

### Defensins and antiviral assays

Mature HD5 and Crp23 were obtained by oxidative refolding of partially purified linear peptides and purifying the correctly folded species by reverse-phase high-pressure liquid chromatography (RP-HPLC) [21]. ProHD5 and HD5Abu were chemically synthesized and purified as described [61, 62]. Crp2 was synthesized, refolded, and purified using the same method as for HD5 [61]. Purified Crp4 was produced in *E*. *coli* and purified by RP-HPLC [63].

For antiviral assays, serial dilutions of MAdV-1.IXeGFP were used to infect CMT-93 cells in black wall, clear bottom 96-well plates (Perkin-Elmer). Total well fluorescence was quantified with a Typhoon 9400 variable mode imager (GE Healthcare) 2 d p.i. Antiviral assays used a virus concentration producing 50–80% maximal signal. To measure their effects on infectivity, increasing concentrations of α-defensins were incubated with purified MAdV-1.IXeGFP for 45 min on ice in serum-free DMEM (SFM). The mixture was then added to a confluent monolayer of CMT-93 cells in 96-well plates that had been washed twice in SFM. Cells were then incubated at 37°C for 2 h with rocking, washed, and cultured with complete DMEM for 2 d. Total well fluorescence was measured by Typhoon, and background-subtracted fluorescence was quantified using ImageJ software [64]. Fluorescence values were compared to control wells infected in the absence of inhibitor.

### Dynamic light scattering

α-defensins were serially diluted in 10 mM Tris, 150 mM NaCl, pH 7.5 and mixed with 6.5 x 10^8^ particles of purified wild type MAdV-1 in a total volume of 50 μl. Control samples of MAdV-1 or α-defensin only were diluted in the same buffer. Samples were incubated for 45 min on ice and then equilibrated for 3 min at 37°C prior to analysis. The z-average particle size was obtained by cumulant analysis with a Malvern Zetasizer Nano ZS and manufacturer’s software (Malvern Instruments).

### Ethics statement

All mouse experiments were performed in strict accordance with the Guide for the Care and Use of Laboratory Animals of the National Institutes of Health and following the International Guiding Principles for Biomedical Research Involving Animals. Experiments were approved by the Institutional Animal Care and Use Committee of the University of Washington under University of Washington Protocol Number 4245–01.

### Animals and MAdV-1 infection studies

Wild type C57BL/6NHsd (Harlan Laboratories) and isogenic *Mmp7* ^*-/-*^ mice [65] were mated to generate progeny mice heterozygous for *Mmp7*. These mice were then intercrossed to generate wild type and *Mmp7* ^-/-^ lines, which were used to breed mice for all experiments over three generations of progeny. Mice were housed under specific pathogen-free conditions and were infected for experiments under ABSL2 conditions between 5 and 9 weeks of age. Mice were infected via oral gavage with 100 μl of sterile tissue culture supernatant containing virus diluted in sterile PBS, except for experiments in Fig 2*C* and 2*D* for which virus was administered intraperitoneally in a volume of 100 μl. For experiments in Figs 2*A*–2*D*, 4*A*, and 4*B*, mice were housed individually beginning 3 or 4 d prior to infection and for the duration of the experiment. For the experiment in Fig 3*B*, mice from both genotypes were co-housed at weaning and for the duration of the experiment, and the genotypes of individual mice were blinded during the study. Mice were humanely euthanized by CO_2_ inhalation if moribund, if partially paralyzed, if seizing, if weight loss from maximal recorded weight exceeded 20%, or at the end of the experiment (28 d p.i.). Food (PicoLab Rodent Diet 5053, LabDiet) and water were provided *ad libitum*. Mice were weighed on the day of infection and every 1–2 d thereafter, as indicated. Percent weight change between measurements was calculated by subtracting the previous weight from the current weight, dividing by the previous weight, and multiplying by 100.

### Commensal depletion

Mice were treated with 100 μl of a mixture containing pharmaceutical grade ampicillin (100 mg/ml, Sandoz), neomycin sulfate (100 mg/ml, PCCA), metronidazole benzoate (100 mg/ml, PCCA), and vancomycin HCl (50 mg/ml, Mylan) in Ora-Sweet Syrup Vehicle (Paddock Laboratories) and peanut butter flavoring (PCCA) by oral gavage for 5 d. Control mice were gavaged with peanut butter-flavored vehicle without antibiotics. Upon initiation of antibiotic treatment and for the duration of the experiment, mice were also given a 100-fold lower concentration of the same antibiotic mixture or vehicle control *ad libitum* in drinking water. Fecal samples (1 pellet/mouse) were collected on day 5 of treatment and periodically thereafter directly from the mouse into sterile PBS and homogenized. The fecal material was serially diluted and cultured on brain heart infusion (Sigma-Aldrich) agar plates supplemented with 10% sheep’s blood (Colorado Serum Company). Plates were cultured anaerobically for 72 h using the GasPak EZ Anaerobe Puch System (BD). Colonies were counted to obtain culturable CFUs with a limit of detection of 50 CFU. After 23 d of continuous antibiotic treatment, mice were infected with MAdV-1 by oral gavage as above.

### Histology

Mice were exsanguinated by cardiac puncture under Avertin anesthesia. To minimize autolysis, organs (MLN, duodenum, ileum, and spleen) were immediately subjected to immersion fixation in 10% neutral buffered formalin for at least 24 h, embedded in paraffin, sectioned at 5 μm, and stained with hematoxylin and eosin (H&E). A board-certified pathologist scored blinded and randomized samples using a previously established scoring system [66]. Selected mice from the experiments in Fig 2 were subjected to a complete diagnostic necropsy, which was not blinded. To classify and count ileal crypts, H&E stained slides were digitized using a Nanozoomer C9600 Whole Slide Scanner (Hamamatsu) and annotated using NDP2.view2 software (Hamamatsu) based on Paneth cell phenotypes visualized by bright field microscopy at higher magnification (400–600x). To visualize germinal centers, serial sections were stained with biotin conjugated peanut agglutinin (PNA) (Vector Labs) after antigen retrieval in citrate buffer pH 6. Bound lectin was detected using the ABC Elite kit (Vector Labs) and visualized with 3, 3-diaminobenzidine (DAB). Sections were counterstained with hematoxylin.

### Quantification of viral genomes in tissue and feces

At the time of necropsy, small (~10 mm^3^) samples of hindbrain (by pinch biopsy), ileum, and spleen were snap frozen in liquid N_2_ and stored at -80°C. DNA was extracted using the DNeasy Blood and Tissue Kit (Qiagen). MAdV-1 genomes were quantified by quantitative PCR (qPCR) using the SsoFast EvaGreen Supermix (Bio-Rad) against a standard curve of pKBS2-MAdV1 using primers M1FF2 (5’-ATTCCATGATACCCGCCTAA-3’) and M1FR2 (5’-TCCAACCAATTCCAGCATAA-3’). Cellular genomes were quantified against a standard curve of C57BL/6 liver DNA (286 genome copies/ng of DNA) using primers MmGRO1F and MmGRO1R [67]. For both reactions, conditions consisted of 40 cycles of PCR with 55°C annealing temperatures using an iCycler (Bio-Rad). Limits of detection were defined by the ratio of viral to cellular gene copies detected in samples from each tissue of uninfected mice.

To obtain a representative sample of the feces produced by each mouse and to minimalize sampling error, fecal samples consisted of ten fecal pellets that accumulated in the cages of single-housed mice since the previous collection. Accordingly, on the day of infection and after every fecal collection, mice were transferred to new cages with clean bedding. Mice from the cohorts to be euthanized on days 9 and 11 p.i. were analyzed. DNA was extracted from fecal samples using the QIAamp DNA Stool Mini Kit (Qiagen) into a total volume of 200 μl. Viral genome copies in 1 μl of this sample were quantified as above and the values (without normalization) were plotted in Fig 4*C*. The limit of detection was defined by the number of viral copies detected in feces from uninfected mice.

### Neutralizing antibody (NAb) assay

Serial dilutions of MAdV-1 were used to infect CMT-93 cells in black wall, clear bottom 96-well plates. Cells were fixed and stained with anti-hexon antibody 8C4 and an Alexa Fluor 488-conjugated secondary antibody, and total well fluorescence was quantified with a Typhoon 2 d p.i., as above. NAb assays used a virus concentration producing 50–80% maximal signal. Two-fold serial dilutions of heat-inactivated (56°C for 1 h) serum were incubated with MAdV-1 for 45 min at RT and added to a confluent monolayer of CMT-93 cells in 96-well plates. After 2 d, cells were stained for hexon, scanned, and background-subtracted fluorescence was quantified using ImageJ software. Fluorescence values were compared to control wells infected in the absence of serum. The greatest dilution of serum that inhibited virus by at least 50% was considered the neutralizing activity of each sample. For Fig 7*A*, serum from each mouse was analyzed in three independent experiments, and the mean titer for each mouse was calculated by averaging the log transformed value of each replicate. Samples for which no inhibition was observed were imputed with the minimum dilution used for the assay (10 in one replicate and 20 in two replicates). For Fig 7*B*, samples were tested once at three serum dilutions.

### Ovalbumin exposure and antibody response

Wild type and *Mmp7* ^*-/-*^ mice were immunized with a sterile mixture of OVA (50 μg/mouse, Sigma-Aldrich) and *E*. *coli* 0111:B4 LPS (5 μg/mouse, Sigma-Aldrich) in PBS via i.p. injection (100 μl/mouse) or intranasal instillation (20 μl/mouse). Intranasal instillation was performed under light isoflurane anesthesia [68]. Each treatment group of 10 animals was divided into two cohorts of 5 mice, which were sampled by submandibular bleed on alternating days. The cohorts were also euthanized on different days to obtain cardiac blood. Thus, serum samples were obtained from each treatment group on days 4, 7, 9, 11, 13, and 15 post-inoculation. An additional wild type mouse was inoculated i.p., boosted i.p. on days 14 and 21, and euthanized on day 28 post-inoculation as a positive control for ELISA. Anti-OVA antibodies were quantified by ELISA using microtest plates (BD Biosciences) incubated overnight with 50 μl/well of 125 μg/mL OVA in coating buffer (0.1 M Na_2_CO_3_, 0.1M NaHCO_3_, pH 9.6) or coating buffer alone. Wells were blocked with ELISA block (5% nonfat dry milk, 0.05% Tween-20, 0.025% NaN_3_, in PBS) for 1 h at 37°C, and heat inactivated (56°C for 30 min) serum diluted 20-fold into ELISA block was incubated overnight at 4°C in OVA-coated and control uncoated wells. Antibodies were detected with a peroxidase-conjugated pan-IgA, IgM, and IgG anti-mouse antibody (Sigma-Aldrich) and developed with TMB Substrate (Pierce); and OVA-specific signal for each mouse (OVA signal minus uncoated control) was normalized to the OVA-specific signal from the serum of the positive control mouse. Each sample was quantified in two independent assays, and the average of the two values for each mouse was plotted in Fig 7*C* and 7*D*.

### Statistical analysis

Experiments were analyzed using Prism 5.0d, and for all tests P<0.05 was considered significant. Survival curves were compared by log-rank (Mantel-Cox) test, using the Bonferroni method for multiple comparisons in Fig 3*B*. For Figs 4, 5, 6 and 7 data were compared using one-way ANOVA with Bonferroni post-tests to compare wild type and *Mmp7* ^*-/-*^ mice at each time point. For Figs 4*D*–4*F* and 7*A*, data were log transformed prior to this analysis.

### Accession numbers

Cryptdin 2 (Gene: Defa2, Species: Mus musculus, UniProtKB: P28309)

Cryptdin 4 (Gene: Defa4, Species: Mus musculus, UniProtKB: P28311)

Cryptdin 23 (Gene: Defa23, Species: Mus musculus, UniProtKB: Q5G866)

Human defensin 5 (Gene: DEFA5, Species: Homo sapiens, UniProtKB: Q01523)

Matrix metalloproteinase-7 (Gene: Mmp7, Species: Mus musculus, UniProtKB: Q10738)

## Supporting Information

S1 Fig Weight change of mice treated with or without antibiotics and infected orally with MAdV-1. Data are weight change from measure to measure for each mouse from Fig 3*B* as follows: (*A*) wild type without antibiotics (Abx), (*B*) wild type with Abx, (*C*) *Mmp7* ^-/-^ without Abx, (*D*) *Mmp7* ^-/-^ with Abx. Open gray symbols containing an “x” denote mice that were humanely euthanized due to illness. Day 0 is the time of oral challenge with MAdV-1 for all graphs.
(TIF)
