# Supplementary figures and images for: Defensins Potentiate a Neutralizing Antibody Response to Enteric Viral Infection
Source: PLoS Pathog. 2016 Mar 2;12(3):e1005474. doi: 10.1371/journal.ppat.1005474 (PMC4774934; doi:10.1371/journal.ppat.1005474)

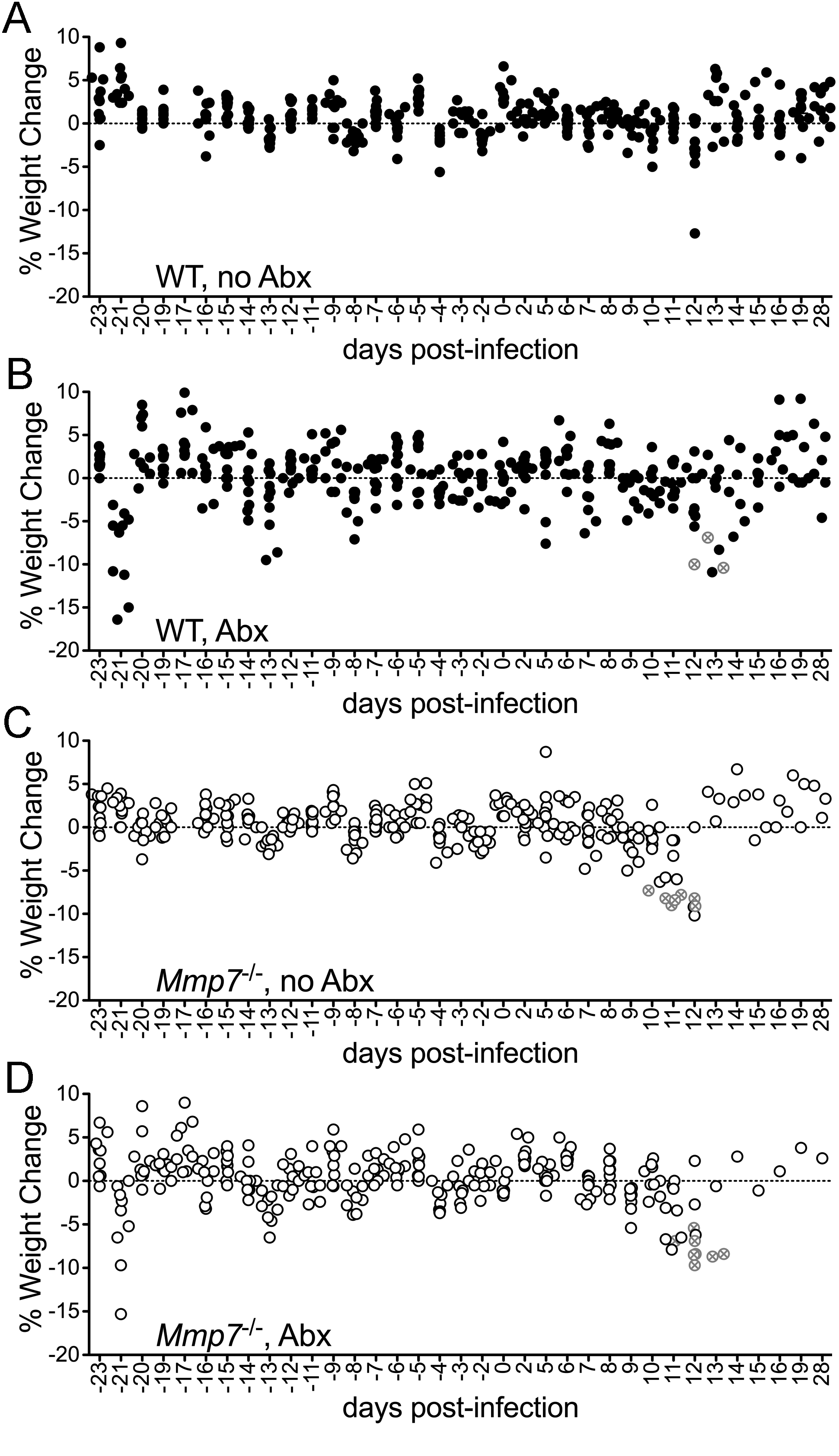

Supplement: S1 Fig — Data are weight change from measure to measure for each mouse from Fig 3B as follows: (A) wild type without antibiotics (Abx), (B) wild type with Abx, (C) Mmp7 -/- without Abx, (D) Mmp7 -/- with Abx. Open gray symbols containing an “x” denote mice that were humanely euthanized due to illness. Day 0 is the time of oral challenge with MAdV-1 for all graphs. (TIF) [file ppat.1005474.s001.tif]
